# Polymorphisms in cytochrome P450 2C19 enzyme and cessation of leflunomide in patients with rheumatoid arthritis

**DOI:** 10.1186/ar3911

**Published:** 2012-07-12

**Authors:** Michael D Wiese, Matthew Schnabl, Catherine O'Doherty, Llewellyn D Spargo, Michael J Sorich, Leslie G Cleland, Susanna M Proudman

**Affiliations:** 1 Sansom Institute for Health Research, University of South Australia, Frome Road, Adelaide, SA 5000, Australia; 2 School of Pharmacy and Medical Sciences, University of South Australia, Frome Road, Adelaide, SA 5000, Australia; 3 Department of Rheumatology, Royal Adelaide Hospital, North Terrace, Adelaide, SA 5000, Australia; 4 School of Medicine, Adelaide University, North Terrace, Adelaide, SA 5000, Australia

## Abstract

**Introduction:**

Rational selection of disease modifying anti-rheumatic drugs in the treatment of rheumatoid arthritis (RA) has many potential advantages, including rapid disease control, reduced long-term disability and reduced overall cost to the healthcare system. Inter-individual genetic differences are particularly attractive as markers to predict efficacy and toxicity, as they can be determined rapidly prior to drug selection. The aims of this study, therefore, were to investigate the association between differences in genes associated with the metabolism, clearance and efficacy of leflunomide with its cessation in a group of rheumatoid arthritis patients who were treated with an intensive contemporary, treat-to-target approach.

**Methods:**

This retrospective cohort study identified all individuals who received leflunomide and were enrolled in the Early Arthritis inception cohort at the Royal Adelaide Hospital between 2001 and July 2011. Inclusion criteria were age (>18) and a diagnosis of rheumatoid arthritis. Patients were excluded if a DNA sample was not available, if they withdrew from the cohort or if clinical data were insufficient. Subjects were followed for 12 months or until either another disease modifying antirheumatic drug was added or leflunomide was ceased. The following single nucleotide polymorphisms (SNPs) were determined: CYP2C19*2 (rs4244285), CYP2C19*17 (rs12248560), *ABCG2 *421C>A (rs2231142), CYP1A2*1F (rs762551) and *DHODH *19C>A (rs3213422). The effects of variables on cessation were assessed with Cox Proportional Hazard models.

**Results:**

Thirty-three of 78 (42.3%) patients ceased leflunomide due to side effects. A linear trend between cytochrome P450 2C19 (*CYP2C19) *phenotype and leflunomide cessation was observed, with poor and intermediate metabolizers ceasing more frequently (adjusted Hazard Ratio = 0.432 for each incremental change in phenotype, 95% CI 0.237 to 0.790, *P *= 0.006). Previously observed associations between cytochrome P450 1A2 *(CYP1A2) *and dihydro-orotate dehydrogenase *(DHODH) *genotype and toxicity were not apparent, but there was a trend for ATP-binding cassette sub-family G member 2 *(ABCG2) *genotype to be associated with cessation due to diarrhea.

**Conclusions:**

*CYP2C19 *phenotype was associated with cessation due to toxicity, and since *CYP2C19 *intermediate and poor metabolizers have lower teriflunomide concentrations, it is likely that they have a particularly poor risk:benefit ratio when using this drug.

## Introduction

Rheumatoid arthritis (RA) is a potentially disabling form of inflammatory arthropathy which displays both articular and systemic manifestations. Disease modifying anti-rheumatic drugs (DMARDs) are the cornerstone of treatment as they suppress underlying immune-mediated inflammation and joint damage and prevent long-term morbidity and mortality. Initial DMARD therapy is typically methotrexate monotherapy or a combination of DMARDs (such as 'triple therapy' with methotrexate, sulfasalazine and hydroxychloroquine [1]). In the case of resistant disease, therapy progresses to leflunomide and/or biological DMARDs, such as tumor necrosis factor (TNF) inhibitors.

Leflunomide is an effective DMARD that was introduced for use principally as a single agent in RA resistant to better established and less expensive DMARD treatments. Nowadays, it is often combined with other DMARDs after failure of initial therapy and is considerably cheaper than biological DMARDs. In contrast to traditional single agent regimens, when leflunomide is used as an adjunct to methotrexate or the other DMARD components of 'triple therapy', lower doses are often used initially and for continuing therapy. Accordingly, there is a need to re-evaluate cessation and continuation rates when leflunomide is used as additional therapy when triple therapy alone has not achieved disease control.

Because tolerance and response to leflunomide may be influenced by polymorphisms in genes which encode proteins involved in the metabolism and clearance of leflunomide and its active metabolite, teriflunomide, we have undertaken an analysis of the effect of candidate polymorphisms on cessation/continuation rates of leflunomide. Polymorphisms were chosen based on the potential importance of proteins for which they encode on steady state teriflunomide blood levels via an effect on leflunomide metabolism (CYP2C19*2 (*CYP2C19 *681G>A, rs4244285) and CYP2C19*17 (*CYP2C19*, 608C>T, rs12248560) [2,3]) or biliary secretion of teriflunomide (*ABCG2 *421C>A, rs2231142) [4,5]). Polymorphisms that had been linked to leflunomide toxicity (CYP1A2*1F, (CYP1A2 163C>A, rs762551) and *DHODH *(*DHODH *19C>A, rs3213422) [3,6,7]) were also studied. *DHODH *was also of interest because inhibition of the encoded protein dihydro-orotate dehydrogenase (DHODH) by teriflunomide appears to be the principal basis of the anti-inflammatory/immunosuppressive effects of leflunomide [8].

Identification of individuals with particularly unfavorable risk:benefit profiles (due to greater risk of toxicity or resistance to therapy) should permit more rational selection of DMARDs by minimizing both unwanted effects and the delay to commencement of potentially more effective, albeit more costly, therapies, such as TNF inhibitors, which ultimately should reduce long term joint damage and associated disability.

In a group of RA patients who were being treated with an intensive, contemporary approach (that is, concurrently with other conventional DMARDs, with dose modification according to a treat-to-target protocol [1]), the primary aim of this cohort study was to investigate the impact of pharmacogenomic factors on the likelihood of cessation of leflunomide after adjusting for the influence of more conventional factors.

## Materials and methods

### RA patient population and treatment

Patients who enrolled in the Early Arthritis inception cohort at the Royal Adelaide Hospital who had taken leflunomide between 2001 and July 2011 were considered for inclusion in this study. Other inclusion criteria were being over 18 and a diagnosis of RA according to the 1987 revised American College of Rheumatology criteria [9]. Patients were excluded from the analysis if there was no DNA sample available for analysis, if clinical records were insufficient or if they withdrew from the cohort study within the follow-up period.

Patients were treated with a standardized protocol that has been described previously [1]. Leflunomide was added after combination treatment had been up-titrated (if tolerated) to the following doses: methotrexate (25 mg sub-cutaneously per week), sulfasalazine (3 g orally daily) and hydroxychloroquine (400 mg orally daily). During the first three years of the cohort study, leflunomide was introduced with a loading dose (three daily doses of 100 mg followed by 20 mg daily), and thereafter it was introduced at a daily dose of 10 mg. Patients were generally reviewed every six weeks while they had active disease, or every three months if disease was inactive. If treatment targets were not met while they were taking a daily leflunomide dose of 10 mg, the dose was increased to 20 mg. Data were collected for 12 months after initiation of leflunomide.

The following information was collected at baseline: age, gender, height, weight, smoking status, time since diagnosis, ethnicity, use of other DMARDs, blood pressure, full blood count, liver function tests, shared epitope, anti-cyclic citrullinated peptide (anti-CCP) antibody and rheumatoid factor titer. At each clinic visit, leflunomide dose was recorded, patients were questioned about side effects to all DMARDs and results of full blood count, blood pressure readings and liver function tests were recorded.

The duration of leflunomide was defined when the patient had either i) ceased leflunomide due to side effects or ii) was continuing treatment (for example, 12 months after initiation of leflunomide) and/or if another DMARD had been added. Leflunomide was deemed to have been ceased due to a side effect as determined by the patient's clinician.

Individuals who continued to take leflunomide beyond 12 months were censored at their next clinic appointment or after 400 days (whichever was earlier), and those who had another DMARD added were not considered to have ceased due to side effects and were censored on the day that the new DMARD was added and included in the analysis.

The study was approved by the research ethics committees of the Royal Adelaide Hospital and the University of South Australia. Patients gave informed written consent to their enrolment in the Royal Adelaide Hospital Early Arthritis inception cohort and for collection of blood samples for DNA extraction and analysis.

### Genotype evaluation

Genomic DNA was extracted from patient peripheral blood mononuclear cells and stored at -80°C until retrieval. The concentration and purity of the samples was determined using a Nanodrop (Thermo Fisher Scientific, Waltham, MA, USA). DNA was plated into a 96-well master plate at 5 ng/μL and stored at -20°C until required.

The following SNPs were determined by TaqMan® SNP genotyping assays using an ABI 7500 Fast Real-Time PCR system (Applied Biosystems, Carlsbad, CA, USA): CYP2C19*2 (rs4244285), CYP2C19*17 (rs12248560), *ABCG2 *421C>A (rs2231142), CYP1A2*1F (rs762551) and *DHODH *19C>A (rs3213422). Assays were carried out according to the manufacturer's instructions.

### Statistical analysis

Statistical analyses were conducted with R Version 2.13 (The R foundation for statistical computing, Vienna, Austria). All analyses of genotypes were adjusted for variables that influenced the rate of cessation due to toxicity. *CYP2C19 *was analyzed on the basis of expected phenotype as per the system of Pare *et al*. [10]. CYP2C19*2 was considered the loss of function allele, while CYP2C19*17 was the gain of function allele. Briefly, poor metabolizers carry two loss of function alleles, intermediate metabolizers carry one loss of function and one wild-type allele, extensive metabolizers carry two wild-type alleles and ultra-rapid metabolizers carry either one or two gain of function alleles and no loss of function alleles. *CYP2C19 *unknown metabolizers carry both a loss and gain of function allele, and were excluded from this analysis. The CYP1A2*1F CC genotype and *CYP2C19 *poor metabolizers had a population frequency <10%, so were pooled with CYP1A2*1F AC genotype and *CYP2C19 *intermediate metabolizers respectively for analysis. Cox proportional hazard models were used to estimate the Hazard Ratio of the association between patient characteristics and time to discontinuation of leflunomide due to side effects. The statistical significance of the Hazard Ratio was assessed using the likelihood ratio test. Proportionality of the Hazard Ratio was assessed using the goodness-of-fit statistic and by inspecting a plot of Schoenfeld residuals over time. A linear trend for effect between *DHODH *genotypes and *CYP2C19 *poor/intermediate, extensive and ultrarapid metabolizers was assessed. Other patient characteristics that influenced the risk of leflunomide discontinuation were included in a multivariate Cox proportional hazards model to adjust for potential confounding and to assess the incremental effect of adding genotype or phenotype to more conventional information. The effect of leflunomide dose was assessed using a time-dependent covariate in the Cox proportional hazards model, which indicated periods in which a leflunomide daily dose of 10 mg or 20 mg was being taken. Adjusted survival curves were estimated using direct adjustment [11].

## Results

From the 252 individuals who had been enrolled into the Early Arthritis inception cohort up to July 2011, 84 were found to fulfil the inclusion criteria, and of these, 78 were included in the analysis. Of the six excluded individuals, three had insufficient clinical data, two withdrew from the cohort study during the follow-up period due to diagnosis of a malignant tumor and one ceased of their own volition due to a side effect unlikely to be caused by leflunomide. Of the two patients who withdrew from the cohort study, one was diagnosed with a primary colorectal tumor which was revealed by investigations undertaken after a change in bowel habit shortly after initiation of leflunomide, and the other was diagnosed with metastatic lung cancer, which was identified after 41 weeks of leflunomide treatment. The baseline characteristics of the 78 included individuals are outlined in Table 1.

**Table 1:** Baseline patient characteristics (n = 78)

Age (years), median (IQR)	56.4 (48 to 66.75)
Body Mass Index (kg/m^2^), median (IQR)	26.6 (24.4 to 30.2)

Female	61 (78.2%)

Ever Smoked	47 (60.3%)

Current smokers	22 (28.2%)

Caucasian race	73 (93.6%)

*Continuing triple DMARD therapy	51 (65.4%)

*Continuing methotrexate	60 (76.9%)

*Continuing sulfasalazine	60 (76.9%)

*Continuing hydroxychloroquine	70 (89.7%)

Initiation of leflunomide with loading dose	7 (9.0%)

Anti-CCP antibody (n = 77)	38 (49.4%)

Rheumatoid factor	46 (59.0%)

Shared epitope (n = 76)	52 (68.4%)

Duration of disease prior to initiation of leflunomide (weeks), median (IQR)	48 (32.3 to 92.1)

DAS28 (at start of leflunomide therapy), median (IQR)	5.8 (5.2 to 6.5)

*- Participants could be included in any or all of these groups.

**Figure 1:**
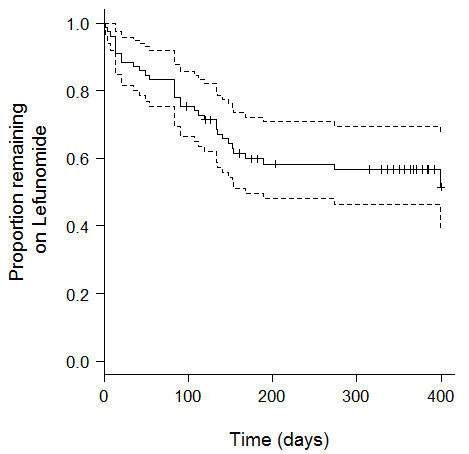
**Rates of discontinuation of leflunomide**. The solid line shows the actual rates of continuation of leflunomide and the dashed lines the 95% confidence interval.

During the follow-up period, a total of 50 patients (64.1%) increased their daily leflunomide dose to 20 mg after a median of 49 days, and 4 patients (5.1%) required addition of another DMARD due to a sub-optimal response to leflunomide. In all, 47 individuals (60.3%) suffered an adverse drug reaction attributable to leflunomide and 33 (42.3%) ceased leflunomide due to side effects. A total of 52 side effects that were responsible for cessation are shown in Table 2, with diarrhea (n = 8), nausea/vomiting (n = 7) and elevated liver transaminases (n = 6) being the most frequently reported. The proportion of patients still taking leflunomide over the duration of the study is shown in Figure 1.

**Table 2:** Side effects leading to cessation of leflunomide

Side effect	Number of patients reporting side effect
Diarrhea	8

Nausea/Vomiting	7

Elevated transaminases*	6

Shortness of breath/cough/pneumonitis	5

Dizziness/fainting	4

Rash	3

Haematological	3†

Abdominal cramps/bloating	3

Hair loss	2

Fatigue	2

9 others‡	1 each

* Elevated transaminases ranged from 2 to 30 times the upper limit of normal

† Includes one case each of oxidative haemolysis, neutropenia and leucopenia

‡ one each of muscle pain, blurred vision, facial flushing, mouth ulcers, taste disturbance, heavy/painful menstruation, peripheral neuropathy, loss of weight and headaches.

Patient characteristics that were significantly associated with an altered rate of cessation were positive anti-CCP antibodies at the time of RA diagnosis (Hazard Ratio = 0.43, 95% CI 0.21 to 0.92, *P *= 0.029) and positive rheumatoid factor at the time of RA diagnosis (Hazard Ratio = 0.49, 95% CI 0.25 to 0.98, *P *= 0.044). As expected, anti-CCP antibody was strongly correlated with rheumatoid factor (*P *<0.001). Other variables that were associated with cessation rate were current use of a 20 mg daily dose (Hazard Ratio = 1.68, 95% CI 0.79 to 3.56) and use of triple DMARD therapy at the time of leflunomide initiation (Hazard Ratio = 2.2, 95% CI 0.97 to 5.2), but these did not reach statistical significance (*P *= 0.18 and 0.057 respectively). There was a trend for a correlation between continuation of triple therapy with anti-CCP antibodies (*P *= 0.079), but not rheumatoid factor (*P *= 0.97), so positive anti-CCP antibodies were not included in the final model.

**Figure 2:**
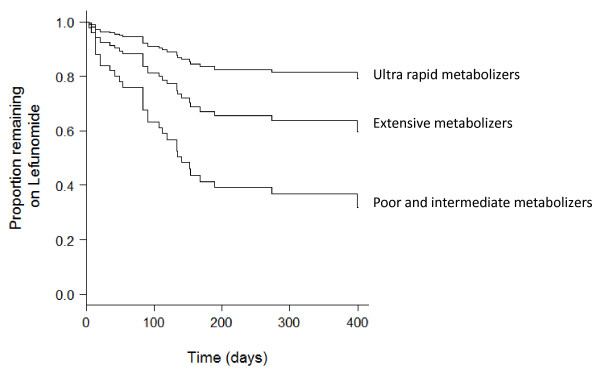
**Cox proportional hazard model to estimate the rates of discontinuation according to *CYP2C19 *phenotype (Hazard Ratio = 0.432 for each incremental change in phenotype, 95% Confidence Interval 0.237 to 0.790, *P *= 0.006 after adjustment for current leflunomide dose, continuation of triple therapy and positive rheumatoid factor at diagnosis)**.

After adjusting for current leflunomide dose, triple therapy and positive rheumatoid factor at diagnosis, *CYP2C19 *phenotype was significantly associated with the likelihood of cessation, with ultra-rapid metabolizers ceasing less frequently than extensive and intermediate/poor metabolizers (Table 3 and Figure 2, Hazard Ratio = 0.432 for each incremental change in phenotype, 95% CI 0.237 to 0.790, *P *= 0.006). As a sensitivity analysis, non-Caucasian subjects were removed from the analysis, and the resultant Hazard Ratio was 0.409 (95% CI 0.219 to 0.762, *P *= 0.005). No association between *ABCG2 *(*P *= 0.572), *CYP1A2 *(*P *= 0.954) or *DHODH *(*P *= 0.993) genotype and leflunomide cessation was apparent (Table 3), but interestingly, it was noted that while 0 of 14 who had the AC genotype at 421C>A *ABCG2 *ceased leflunomide as a result of diarrhea (+/- other side effects), 8 of the 64 patients with the 421*CC genotype (12.5%) ceased treatment for this reason (*P *= 0.198).

**Table 3:** Association of phenotypes and genotypes with cessation due to side effects

SNP	Phenotype/Genotype	Proportion ceased due to side effects (%)	Hazard Ratio (95% Confidence Interval), *P-v*alue*
CYP2C19 (CYP2C19*2 (rs4244285), CYP2C19*17 (rs12248560))	Poor/Intermediate metabolizers	8/15 (53.3%)	0.432 (0.237 to 0.790), *P *= 0.006†
		
	Extensive metabolizers	18/39 (46.2%)	
		
	Ultra-rapid metabolizers	5/21 (23.8%)	

*ABCG2 *421C>A (rs2231142)	AC	6/14 (42.9%)	1.297 (0.527 to 3.194), *P *= 0.572
		
	CC	27/64 (42.2%)	

CYP1A2*1F (rs762551)	AA	16/38 (42.1%)	1.021 (0.508 to 2.050), *P *= 0.954
		
	AC +CC	17/40 (42.5%)	

*DHODH *19C>A (rs3213422)	AA	9/20 (45%)	1.002 (0.610 to 1.648), *P *= 0.993†
		
	AC	16/39 (41.0%)	
		
	CC	8/19 (42.1%)	

All genotypes are in Hardy-Weinberg equilibrium. *after adjustment for dose, continuing triple therapy and positive rheumatoid factor. † Hazard Ratio and *P-*value represent a linear test for trend. The Hazard Ratio refers to the hazard associated with each incremental change in phenotype.

## Discussion

Our results have shown a significant association between *CYP2C19 *phenotype and cessation of leflunomide due to toxicity in a group of patients with RA, most of whom were concurrently receiving other DMARDs.

The CYP2C19*2 loss-of-function allele (carried by poor, intermediate or unknown metabolizers) has been associated with lower teriflunomide concentration, which is thought to be due to reduced conversion of leflunomide to teriflunomide [3]. If the side effects from leflunomide were solely caused by teriflunomide, we would expect intermediate/poor metabolizers to have a lower incidence of toxicity. This was not the case in this study, and suggests either direct toxicity by leflunomide, or an alternate pathway is competing with the conversion of leflunomide to teriflunomide and results in formation of another metabolite which contributes to side effects. The latter is supported by studies of leflunomide in cultured hepatocytes, which indicated that Cytochrome P450 mediated metabolism is a prerequisite for leflunomide induced liver injury, whereas teriflunomide is directly, albeit less significantly toxic [12]. Metabolites of teriflunomide have not been observed in human plasma, but studies with human liver microsomes have shown that teriflunomide can be converted via hydroxylation to a metabolite (M1), whereas leflunomide can be converted to either teriflunomide or a different metabolite (M2, also by hydroxylation) [13,14]. If the enzymes responsible for formation of M2 are different from the well characterized conversion pathway of leflunomide to teriflunomide, reduced activity of CYP2C19 is likely to result in increased formation of M2, which may be the toxic metabolite contributing to the side effects associated with leflunomide.

While we provide no direct evidence for either the formation of M1 or M2 *in vivo *or toxicity associated with M2, we have demonstrated that *CYP2C19 *phenotypes with reduced activity were associated with higher rates of toxicity. Furthermore, since there has been an association between greater reduction in RA disease activity in patients with higher plasma teriflunomide steady state concentration [3,15,16], *CYP2C19 *intermediate/poor metabolizers may have an especially adverse risk:benefit ratio by virtue of a higher risk of cessation with side effects and a reduced likelihood of therapeutic benefit. In contrast, ultra-rapid metabolizers appear to have a particularly favorable risk:benefit ratio. This may be an important factor for clinicians to consider when they are contemplating which DMARD to prescribe their patients with RA. This finding may also explain why there has been no success in identifying a relationship between adverse drug events and teriflunomide concentration.

A number of factors are considered by both physician and patient when deciding whether to cease any medication. A novel feature of our study protocol is that a DMARD is never stopped or switched for reason of inefficacy, and the only reason to cease a DMARD is for toxicity. However, we must acknowledge that both patient and physician perceptions of side effects may be subjective, and we can not exclude the possibility that inefficacy may have influenced withdrawals. To this end, we did not observe any association between response to leflunomide after three months of treatment and *CYP2C19 *phenotype (data not shown), suggesting that reduced efficacy is unlikely to be responsible for increased drug cessation in *CYP2C19 *intermediate and poor metabolizers.

van Roon and colleagues have shown that when leflunomide was given primarily as monotherapy to long term RA patients, 29% withdrew due to an adverse event and 13% ceased due to inefficacy after a median follow-up of 317 days [17]. This compares to a withdrawal rate due to adverse events of 42.3% in our study and addition of another DMARD was required in 5.1% of patients. Since our study had a shorter mean duration from diagnosis to introduction of leflunomide (1.4 vs 9.5 years) and more frequent use of combination DMARDs, the greater efficacy and poorer tolerability observed in our cohort is to be expected and entirely consistent with this previous report [17].

The results of our study are in contrast to previous studies of leflunomide toxicity that have reported no relationship with *CYP2C19 *genotype, but did demonstrate an association with SNPs in *CYP1A2 *and *DHODH *[6,7]. There are a number of important differences in both study design and the participants included in our study compared to these previous reports (which were presumably performed on the same patient population) which may account for these discrepancies. First, our study was a retrospective cohort design, which has advantages over retrospective case control studies, which are prone to additional selection/recruitment bias. The previous study reported that only 19% of the group who had not ceased leflunomide were taking another DMARD (most of whom were also taking methotrexate), whereas the number who ceased leflunomide while taking other DMARDs was not reported; such a discrepancy could account for the differing rates of toxicity between genotypes, which is supported by our finding of higher cessation rates in individuals who were continuing triple therapy. Despite not accounting for these important variables, the authors adjusted for factors that did not appear to influence toxicity, such as age, body mass index (BMI) and disease duration. Our approach of adjusting only for variables that were seen to influence cessation due to toxicity is more robust. Finally, by comprehensively assessing information about multiple *CYP2C19 *genotypes, we were able to consider the entirety of the information regarding CYP2C19 activity concurrently by comparing outcomes related to *CYP2C19 *phenotype instead of each genotype separately. This is an important point of difference with previous studies, since 20 to 25% of individuals who carry a loss (*2) or gain (*17) of function allele are unknown metabolizers and should, therefore, not be considered equivalent to intermediate or ultra-rapid metabolizers respectively. As such, compared to *CYP2C19 *phenotypes, the effect size observed with individual genotypes is likely to be lower and significant associations are, therefore, more difficult to demonstrate.

The conversion of leflunomide to teriflunomide is mediated by CYP1A2 (in addition to CYP2C19) [2], and the immunosuppressive properties are mediated by inhibition of DHODH [8], but there is currently no biologically plausible explanation for the proposed associations of *CYP1A2 *and *DHODH *genotype and leflunomide toxicity [6,7]. The CYP1A2*1F allele occurs in the promoter region of the *CYP1A2 *gene (in intron 1), and is thought to be important in induction of enzymatic activity in smokers (smokers who carried one or two C residues had higher enzyme activity compared to those who had the AA genotype), but it does not have an effect on enzymatic activity in non-smokers [18]. If this mechanism is responsible for the higher rate of adverse events, one would expect that this SNP would only have an effect in current smokers. Given that the current smoking rate in our cohort (28%) was higher than that reported by Bohanec Grabar *et al*. (18%) [6] a more pronounced effect on toxicity would have been expected in our study, but this was not the case. Smokers who carry an AC or CC genotype are likely to be more efficient at converting lefunomide to teriflunomide, but this does not translate to higher teriflunomide concentrations [3]. Furthermore, given the association between *CYP2C19 *phenotype and toxicity suggests that intolerance may be due to a toxic metabolite, enhanced CYP1A2 activity (and, therefore, enhanced conversion of leflunomide to teriflunomide) is more likely to be protective of side effects. The SNP in the *DHODH *gene associated with toxicity by Grabar *et al*. (rs3413422) is associated with an amino acid substitution in the expressed protein (Lys7Gln) [7], but the effect of this on the activity of the expressed DHODH protein is not known. While there is currently no established biological plausibility to support the association of *DHODH *genotype and toxicity, this could change if the functional significance of this polymorphism is determined.

Although it was not statistically significant, reduced activity of the ABCG2 drug efflux pump appeared to be protective against diarrhea. This is despite individuals with reduced ABCG2 activity having higher plasma teriflunomide concentrations, which is likely due to less hepatobiliary recycling and fecal elimination [19]. Reduced secretion of teriflunomide into the gastrointestinal tract may be protective against diarrhea through reduced local effect of teriflunomide on the bowel wall.

Our study did have some weaknesses that must be acknowledged, including the retrospective design, the assessment of patient compliance was limited to direct questioning of study participants and one cannot be certain that all adverse events attributed to leflunomide were both drug related and caused by leflunomide (as opposed to one of the other DMARDs the patient was taking concurrently). Also, where a patient ceased leflunomide due to more than one side effect, no attempt was made to ascertain the relative severity of each side effect (and, therefore, the one primarily responsible for cessation). The small patient numbers also did not allow us to make definitive findings regarding the factors associated with events that occurred at low frequency within the cohort, such as the influence of *ABCG2 *genotype on cessation due to diarrhea.

We are not aware of any previous observations that have linked side effects to any DMARD (including leflunomide) and anti-CCP antibody and rheumatoid factor positivity. However, it is notable that only a proportion of cases that conform to diagnostic or classification criteria for RA express these antibodies [9]. Accordingly, RA may be regarded as a clinical syndrome, in which a substantial proportion of cases have an autoimmune aetiology, as evidenced by the presence of anti-CCP antibodies (and rheumatoid factor with which it is strongly correlated) in serum and synovial fluid and citrullinated peptides within affected joints [20]. Cases that present with the RA syndrome but lack anti-CCP antibodies and rheumatoid factor presumably have other etiologies that, in contrast to sero-positive RA, may be less dependent upon lymphocyte proliferation to sustain a chronic, pathological antigen-specific immune response. The primary mechanism of action of teriflunomide is via inhibition of DHODH, which prevents activated T-lymphocytes from accumulating sufficient pyrimidines to support DNA synthesis, resulting in decreased cell proliferation [14]. Since methotrexate may also compromise the DHODH synthetic pathway [21], it is conceivable that sero-positive RA is particularly responsive to leflunomide, especially when given as an adjunct to methotrexate therapy. In addition to the overlapping effects of methotrexate and leflunomide on pyrimidine synthesis, sulfasalazine and leflunomide each have inhibitory effects on nuclear factor-κB [22,23]. These interactive effects suggest that even lower doses of leflunomide than those used in trials hitherto may yield benefit and be better tolerated than current standard doses, especially in sero-positive RA.

Informed by our observations, future research should investigate whether leflunomide toxicity is mediated by a toxic metabolite, and if so, seeking to identify and quantify the putative toxic metabolite in patient plasma as a basis for defining correlations with toxicity. Also, a prospective evaluation of the factors that influence leflunomide toxicity, which also incorporates measurement of teriflunomide plasma concentrations would seem to be warranted. Such a trial would ideally have higher patient numbers through recruitment from multiple centers that utilize structured DMARD regimens. The value of *ABCG2 *genotype in predicting diarrhea should also be assessed in a larger patient group.

## Conclusions

In our group of 78 patients, most of whom were taking triple DMARD therapy in combination with leflunomide, the *CYP2C19 *phenotype was associated with cessation due to toxicity and altered the risk:benefit ratio when using this drug. The choice of leflunomide may, therefore, be guided further by knowledge of *CYP2C19 *phenotype.

## Abbreviations

Anti-CCP: anti-cyclic citrullinated peptide; CYP1A2: cytochrome P450 1A2; CYP2C19: cytochrome P450 2C19; DHODH: dihydro-orotate dehydrogenase; DMARD: Disease Modifying Anti-Rheumatic Drug; RA: rheumatoid arthritis; SNP: single nucleotide polymorphism; TNF: tumor necrosis factor.

## Competing interests

The authors declare that they have no competing interests.

## Authors' contributions

MDW, MS, CO, LGC and SMP were primarily responsible for study design. Data collection was performed by MS, LDS, LGC and SMP. Data analysis was primarily performed by MDW, MS, CO and MJS. All authors helped to draft the manuscript and have read and approved the final manuscript.
